# Life table analysis of *Anopheles balabacensis*, the primary vector of *Plasmodium knowlesi* in Sabah, Malaysia

**DOI:** 10.1186/s13071-022-05552-9

**Published:** 2022-11-24

**Authors:** Tock H. Chua, Benny Obrain Manin, Kimberly Fornace

**Affiliations:** 1 Department of Pathology and Microbiology, Faculty of Medicine and Health Sciences, Universiti Malaysia Sabah, Sabah, Malaysia; 2 School of Biodiversity, One Health and Veterinary Medicine, University of Glasgow, Glasgow, UK; 3 Saw Swee Hock School of Public Health, National University of Singapore, Singapore, Singapore

**Keywords:** Age-stage two-sex life table, *Anopheles balabacensis*, *Plasmodium knowlesi*

## Abstract

**Background:**

*Plasmodium knowlesi* has become a major public health concern in Sabah, Malaysian Borneo, where it is now the only cause of indigenous malaria. The importance of *P. knowlesi* has spurred on a series of studies on this parasite, as well as on the biology and ecology of its principal vector, *Anopheles balabacensis*. However, there remain critical knowledge gaps on the biology of *An. balabacensis*, such as life history data and life table parameters. To fill these gaps, we conducted a life table study of *An. balabacensis* in the laboratory. Characterising vector life cycles and survival rates can inform more accurate estimations of the serial interval, the time between two linked cases, which is crucial to understanding and monitoring potentially changing transmission patterns.

**Methods:**

Individuals of *An. balabacensis* were collected in the field in Ranau district, Sabah to establish a laboratory colony. Induced mating was used, and the life history parameters of the progeny were recorded. The age-stage, two-sex life table approach was used in the analysis. The culture conditions in the laboratory were 9 h light:15 h dark, mean temperature 25.7 °C ± 0.05 and relative humidity 75.8% ± 0.31.

**Results:**

The eggs hatched within 2 days, and the larval stage lasted for 10.5 days in total, with duration of instar stages I, II, III and IV of 2.3, 3.7, 2.3, 2.2 days, respectively. The maximum total fecundity was 729 for one particular female, while the maximum female age-specific mean fecundity (*m*_*x*_) was 142 at age 59 days. The gross reproductive rate or number of offspring per individual was about 102. On average, each female laid 1.81 ± 0.19 (range 1–7) batches of eggs, with 63% of the females producing only one batch; only one female laid six batches, while one other laid seven. Each batch comprised 159 ± 17.1 eggs (range 5–224) and the female ratio of offspring was 0.28 ± 0.06. The intrinsic rate of increase, finite rate of increase, net reproductive rate, mean generation time and doubling time were, respectively, 0.12 ± 0.01 day^−1^, 1.12 ± 0.01 day^−1^, 46.2 ± 14.97, 33.02 ± 1.85 and 5.97 days.

**Conclusions:**

Both the net reproductive rate and intrinsic rate of increase of *An. balabacensis* are lower than those of other species in published studies. Our results can be used to improve models of *P. knowlesi* transmission and to set a baseline for assessing the impacts of environmental change on malaria dynamics. Furthermore, incorporating these population parameters of *An. balabacensis* into spatial and temporal models on the transmission of *P. knowlesi* would provide better insight and increase the accuracy of epidemiological forecasting.

**Graphical Abstract:**

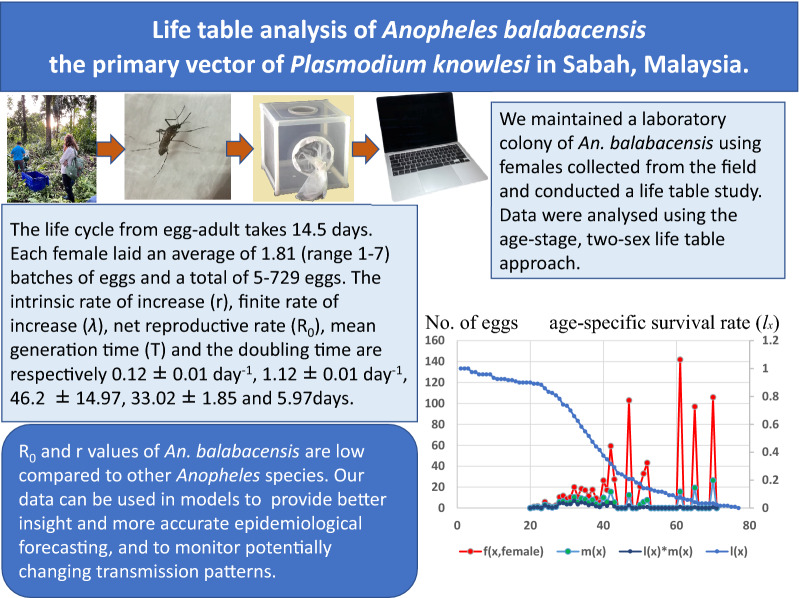

## Background

In Southeast Asia, at least five species of simian malaria parasites, namely *Plasmodium coatneyi*, *Plasmodium* *inui*, *Plasmodium* *fieldi*, *Plasmodium* *cynomolgi* and *Plasmodium* *knowlesi*, have been reported [1, 2], and especially from the primary reservoir, the long-tailed macaque (*Macaca fascicularis*). Among these species, *P. knowlesi* was the first to be recorded infecting humans naturally, in 1965 [3]. In 2004, a large focus of naturally acquired *P. knowlesi* infections in humans was reported in Kapit, Sarawak [4]. *P. knowlesi* is currently the prevalent cause of clinical malaria in Sabah, Malaysian Borneo, where it has become a major public health concern [5, 6]. Another species, *P*. *cynomolgi*, first described by Mayer in 1907 from *Macaca fascicularis* (then known as *Macaca cynomolgus*) imported from Java, had only been reported previously to infect humans accidentally [7]. However, cases of natural infection have now been reported in peninsular Malaysia [8], northern Sabah [9], and Kapit district in Sarawak, Malaysian Borneo [10]. The possibility of *P. cynomolgi* becoming more important in the future cannot be underestimated.

In Sabah state, *P. knowlesi* has now been acknowledged as the most important malaria species, as it is presently the only cause of indigenous malaria there. The increase in the proportion of *P. knowlesi* among indigenous malaria cases, rising from 80% in 2015 to 88% in 2016, and then again to 98% in 2017 of all malaria admissions in the state, is alarming [11]. Furthermore, the proportion of *P*. *knowlesi* of all malaria cases in Sabah increased yearly from 2014 to 2018, i.e. 0.66 (2584/3925), 0.71 (1640/2323), 0.69 (1600/2318), 0.88 (3614/4114) and 0.89 (4131/4630) in each respective year [12]. In 2020, there were zero cases of human malaria in Malaysia for the third consecutive year, but there were 2607 cases of *P. knowlesi* [13]. The male gender was found to be associated with increased risk of symptomatic *P. knowlesi* infection [14]. However, a study on the serological exposure of a large number of subjects in endemic areas showed that, at the community level, patterns of infection and exposure differ markedly with respect to the demographic of reported cases, with higher levels of exposure among women and children [15].

The importance of *P. knowlesi* has spurred on a series of studies on this parasite, as well as on the biology and ecology of its principal vector, *Anopheles balabacensis* [16–18]. Recent studies in Sabah, where land use changes have occurred such as the opening up of forested areas for commercial plantations, and logging, have indicated a clear link between land use change and *P*. *knowlesi* incidence in the region [19, 20]. These results strongly confirm that an epidemiological change is taking place, as previously observed [21]. However, it remains unknown whether this is due solely to increased zoonotic spillover or to changes in transmission patterns resulting in non-zoonotic *P. knowlesi* transmission [22].

Recent research on *An. balabacensis* has shown it to be exophagic, with its major biting time between 6 and 9 p.m., and that it has the potential to infect humans in the peridomestic environment. It is the dominant *Anopheles* species in all habitats, and especially the forest edge, and it prefers to bite humans rather than monkeys if given the choice [17, 18, 23, 24]. Its parity rate varies between 58 and 65%, and its vectorial capacity is 3.9. The gametocytes of *P. knowlesi* take 10 days to develop into transmission stage sporozoites in the female mosquitoes, and those surviving the 10 days have a further life expectancy of 6–8 days [18]. As *An. balabacensis* is found in greater numbers in forest edge habitat compared to human settlements, exposure to this mosquito and its associated zoonoses may be greater for people entering the former habitat [25]. Furthermore, at a community level, the highest probability of human *P. knowlesi* exposure is in areas close to both secondary forest and houses, which indicates the importance of ecotones with respect to exposure to this parasite [15].

There have been few life table studies on *Anopheles* [26–30]. Furthermore, for *An. balabacensis*, there remain critical knowledge gaps regarding its life cycle. Characterising vector life cycles and survival rates can critically inform more accurate estimations of the serial interval, the time between two linked cases [31]. This is crucial to understanding and monitoring potentially changing transmission patterns.

We conducted a laboratory study on the demographic parameters of *An. balabacensis*. Life table data can provide critical information to understand the growth of this vector population in the field. Additionally, these data can inform mathematical models of *P. knowlesi* transmission and provides essential parameters to evaluate the likelihood of two human *P. knowlesi *cases being linked based on the time of reporting.

## Methods

### Establishing and maintaining the colony of *An. balabacensis*

Female *Anopheles balabacensis* were captured from the field in Ranau district, Sabah, using the human landing catch method after ascertaining that the participants had taken prophylactic measures (ethical approval MOH-NMRR-12-786-13,048). The mosquitoes were placed into individual plastic vials (2 cm diameter × 5 cm long, lined with wet tissue paper on the bottom) and taken to the laboratory. The specimens were then transferred to plastic containers (adult container; 8.5 cm diameter × 12 cm high, lined with wet paper towels at the bottom, with the top covered with a piece of wet cloth) and kept overnight in the insectary.

The next day, 25 individuals were isolated randomly to start a colony. The mosquitoes were transferred individually to polystyrene cups filled with 70 mL mineral water that were then covered with a piece of mosquito net. The cups were checked daily to see if the females had laid any eggs. Eggs were transferred to a hatching cup (9 cm diameter × 5 cm high) filled with 150 mL mineral water. The cups were placed under a table lamp (25 W) until the eggs hatched. The identity of the parent (F_0_) was confirmed by morphological characters using published keys [32] and by mitochondrial DNA analysis using a polymerase chain reaction assay [23].

The larvae were collected in groups of 30. Each group of larvae was kept in a hatching cup and fed twice daily (morning and evening). Larvae at stages I and II were fed with 10 mg and 20 mg crushed fish pellets, respectively, and those at stages III and IV were fed with 30 mg and 40 mg crushed fish pellets, respectively. The rearing water was changed every day. The pupae were transferred to pupa containers (4 cm diameter × 5.5 cm high, filled with 70 mL mineral water) within an adult container until the adults emerged. When the adults emerged, groups of 30 were transferred to a new adult container containing a tube of 10% sugar solution that was replaced every 5 days. The age and sex of each adult *An. balabacensis* was recorded.

### Induced mating of *An. balabacensis*

As the mosquitoes did not mate naturally under the laboratory conditions, induced mating [33] was carried out, using randomly selected individuals of both sexes aged 6 days old. First, the male was anaesthetized by exposing it to ether for 15 s. Then, after removing the legs and wings, it was pinned through one side of the thorax using a 15-mm micro pin attached to a wooden stick. The female was also anaesthetized by exposing it to ether for 40 s. When the male had recovered, it was brought close to the anaesthetized female for copulation. Induced mating was considered successful if the female could be lifted up for more than 2 min with the male still attached to her. Each male was used for one mating only. The mated female was allowed to feed on one of our exposed arms, then transferred to an oviposition cup and monitored daily for oviposition. The female was blood-fed every 3–4 days. The eggs laid in the cup were counted and recorded for each female.

Each batch of eggs of a female was transferred to a hatching cup and placed under a table lamp until hatching. The number of eggs that hatched was recorded, and 30 larvae from each batch were collected randomly and reared to the next generation in a plastic container filled with 150 mL mineral water. The number of adult males and females obtained from each batch of eggs was recorded.

The culture room conditions were 9 h light:15 h dark, 25.7 °C ± 0.05 mean temperature and 75.8% ± 0.31 relative humidity. The plastic container conditions were 24.9 °C ± 0.04 mean temperature and 100.0% ± 0.00 relative humidity.

### Hatching rate of *An. balabacensis* eggs

The eggs laid by six randomly chosen females were used for the study. A total of 27 groups, each comprising 20 eggs, were randomly selected. Each group of eggs was placed in a hatching cup, kept under a table lamp for 3 days, and the number of larvae hatching from the eggs recorded.

### Survival rate of *An. balabacensis* larvae

Five females were randomly chosen and their first-instar larvae used for the study. A total of 19 groups, each comprising 20 larvae, were randomly selected and reared until the adult stage. Observations were made daily and the number of larvae surviving or moulting into the next stage was recorded.

### Longevity of *An. balabacensis* adults

A total of 270 adult *An. balabacensis* (sex ratio 1:1) were randomly selected for the study in groups of 10. Five males and five females that emerged on the same day were kept in an adult container until all the adults had died. A 10% sugar solution, replaced every 5 days, was available in the container as food. Observations were carried out daily to record the number of adults that were still alive.

### Data analysis

The raw life history data for *An. balabacensis* were analysed using the age-stage, two-sex life table approach of Chi [34] and Huang and Chi [35]. TWOSEX-MSChart 2020 software (http://140.120.197.173/Ecology/prod02.htm) was used to calculate each parameter, using the following formulae:

(i) ${\mathrm{S}}_{\mathrm{xj}}= \frac{{n}_{xj}}{{n}_{01}}$ which is the age-stage–specific survival rate i.e. the probability of an individual of age *x* and stage *j* surviving to age *x*_*j*_ and stage *j*; n_01_ = daily newborns
(ii) *f*_*xj*_, the age-stage-specific fecundity or the daily number of eggs laid by an individual of age *x* and stage *j*;
(iii) *m*_*x*_, the age-specific fecundity and
(iv) ${l}_{x} = \sum_{j=1}^{m}{S}_{xj}$ which is the age-specific survival rate or the probability that a newly oviposited egg will survive to age *x*.

In addition, the following population parameters were generated for each population from the program. The gross reproductive rate (GRR) was calculated as GRR = ∑*m*_*x*_. The net reproductive rate (*R*_*0*_) is defined as the average number of offspring a female individual produces in her lifetime, and is calculated by ${R}_{0}= \sum_{x=0}^{\infty }{l}_{x}{m}_{x}$. The intrinsic rate of increase (*r*), defined as the number of progeny born to each female mosquito per unit of time, was estimated from the Euler-Lotka equation $\sum_{x=0}^{\infty }{e}^{-r\left(X+1\right)}{l}_{x}{m}_{x}$. The finite rate of increase (*λ*) was obtained as *e*^*r*^. The mean generation time (*T*) was estimated from the formula *T* = (ln*R*_*0*_)/*r*, and defined as the time required for a population to increase to *R*_*0*_-fold its population size at the stable stage of distribution. The TWOSEX-MS Chart program also provided estimated values of the means, SEs and variances of the population parameters using the bootstrap method with 100,000 repetitions. Microsoft Excel (version 16) was used to create graphs.

## Results

### Survivorship, development and longevity

**Fig. 1:**
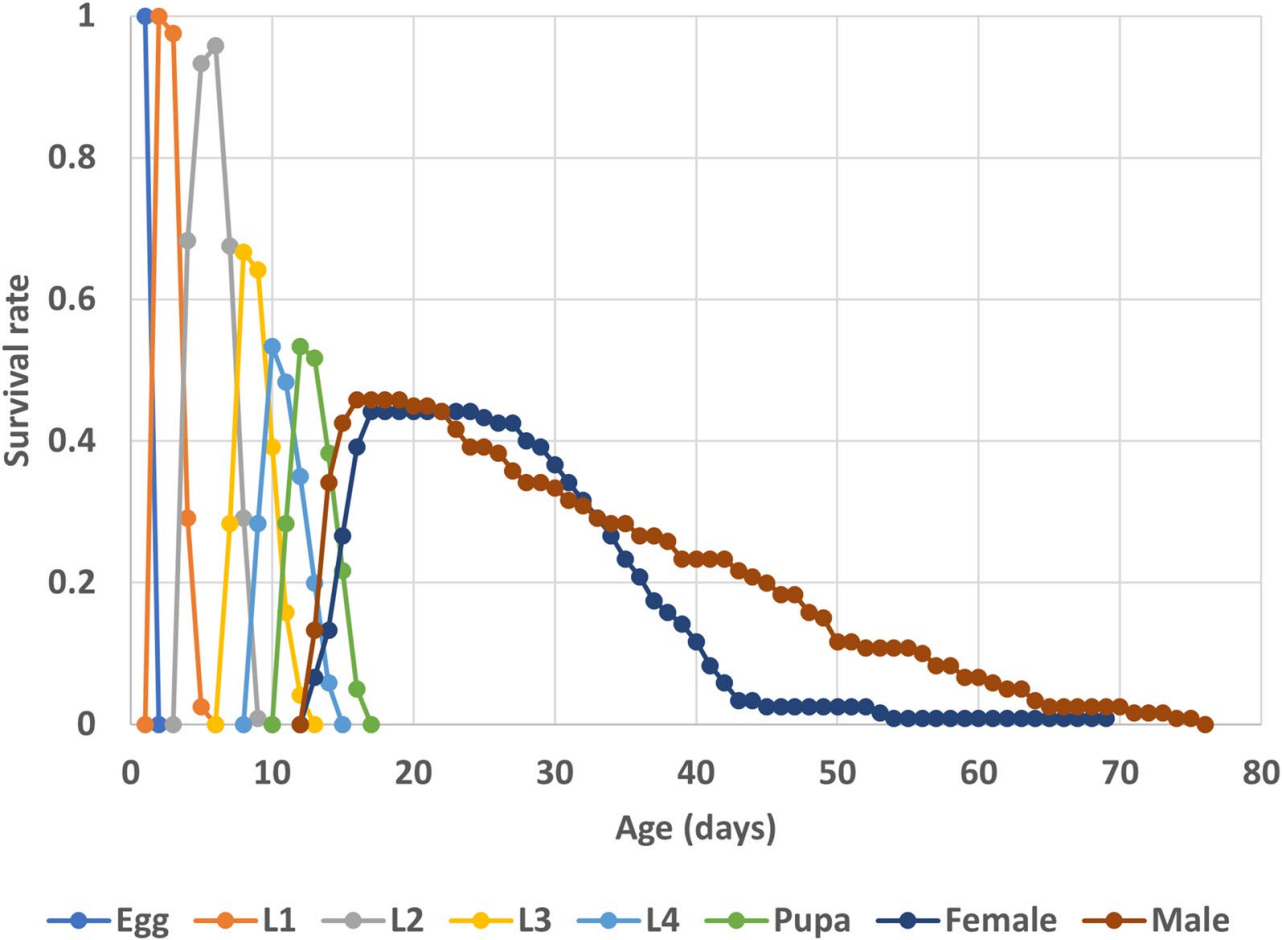
Survival rate (*S*_*xj*_) of *Anopheles balabacensis* reared in the laboratory, where *S*_*xj*_ is the probability that a newly laid egg will survive to age *x* and stage *j*.* L* Larval stage

The eggs hatched within 2 days, and the total development time from egg to final larval stage lasted 12.5 days, with instar duration for stages I, II, III and IV lasting 2.3, 3.7, 2.3, 2.2 days, respectively (Table 1). Under the laboratory conditions, the survival of the immature stages was high, ranging from 0.96 to 1. The pupal stage lasted for about 2 days. Both females and males lived for about 42 days under the laboratory conditions, although the males had a greater range of longevity (4–62 days). Figure  1 shows the plotted age-stage, two-sex life table data on *An. balabacensis* development and stage differentiation.

**Table 1:** Duration (mean ± 95% SE) and survival rate of each developmental stage (*n* = 188) of *Anopheles balabacensis* reared in the laboratory

Stage	Duration (days)	Survival rate
Egg	2.00 ± 0.00	1.0
First instar	2.32 ± 0.05	0.98
Second instar	3.69 ± 0.06	0.98
Third instar	2.32 ± 0.05	0.97
Fourth instar	2.19 ± 0.03	0.99
Egg and larval stages	12.39 ± 0.12	—
Pupa	2.19 ± 0.04	0.98
Female adult longevity	41.72 ± 1.04 (11–54, *n* = 53)	
Male adult longevity	42.44 ± 2.02 (4–62, *n* = 55)

### Fecundity

**Fig. 2:**
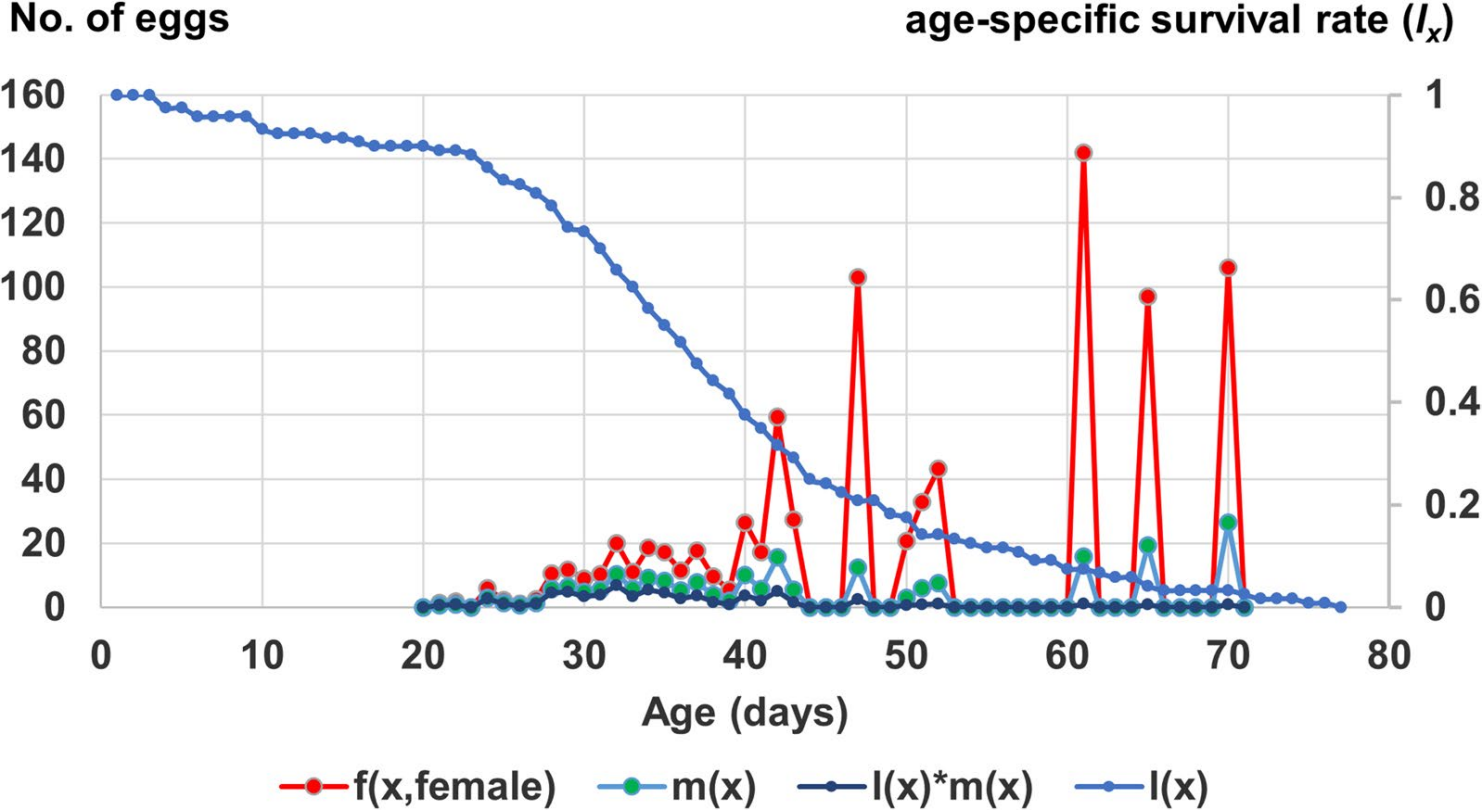
Age-specific survival rate (*l*_*x*_), age-stage fecundity of females (*f*_*ij*_), age-specific fecundity of the cohort (*m*_*x*_) and age-specific maternity (*l*_*x*_*m*_*x*_) of *Anopheles balabacensis*  reared in the laboratory

The adult pre-oviposition period was about 16 days, while the total pre-oviposition period [i.e. the time interval from the birth of a female individual (from the egg stage) to its first oviposition] was 31 days (Table 2). The oviposition days or the mean number of days that a female laid eggs was only about 2 days. The age of the youngest adult recorded for the first oviposition was 20 days, while the oldest female at oviposition was 69 days old. The maximum total fecundity was 729 eggs, for one particular female, while the maximum female age-specific mean fecundity (*m*_*x*_) was 142 eggs at age 59 days, which includes the duration of the pre-adult stage of 16 days. The GRR (offspring per individual) was about 102. On average, each female laid 1.81 ± 0.19 (range 1–7) batches of eggs; 63% of the females in the study laid one batch only, while only one female laid six batches, and only one female laid seven batches. Each batch comprised 159 ± 17.1 eggs (5–526). As the egg batches were laid at discrete time intervals, the peaks (six in total) of female fecundity (*f*_*ij*_) could be observed (Fig. 2).

**Table 2:** Reproduction parameters of *Anopheles balabacensis* reared in the laboratory (mean ± SE)

Reproduction parameters	
Adult pre-oviposition period^a^ (days)	15.55 ± 0.63
Total pre-oviposition period^b^ (days)	30.52 ± 0.63
Oviposition days^c^ (days)	1.94 ± 0.43
Female ratio of offspring	0.28 ± 0.06
Gross reproductive rate (no. of offspring per individual)	102.29 ± 63.60

^a^Period between the emergence of an adult female and her first oviposition

^b^Time interval from birth to the beginning of oviposition

^c^Parameter used to describe fecundity which indicates the number of days on which eggs were laid

### Mortality rates and life expectancy

The stage-specific mortality rates (or the probability that a newborn will die at stage j) are 0% (egg), 2.5% (stage I larva), 1.7% (stage II larva), 3.3% (stage III larva), 0.8% (stage IV larva), 1.7% (pupa), 55.8% (female) and 54.2% (male). The daily survival rate at the adult stage ranged from 0.008 to 0.44 for females and 0.008–0.46 for males, with the rates decreasing with age.

**Fig. 3:**
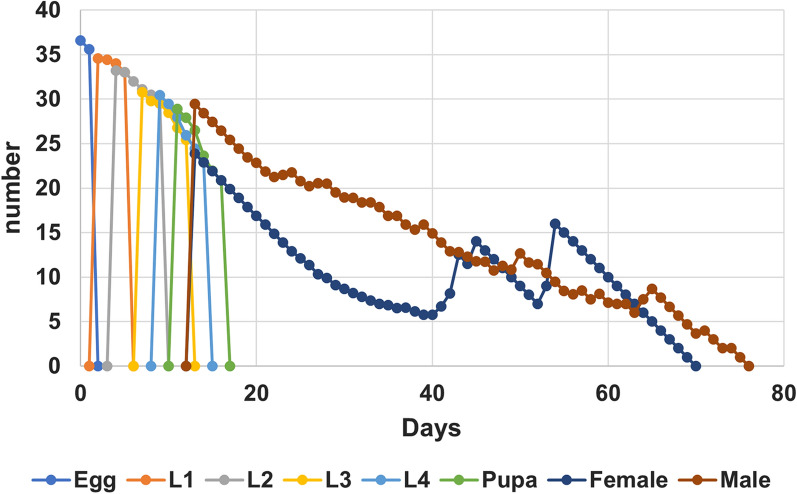
Age-stage-specific life expectancy (*e*_*xj*_; the survival probability of an individual of age *x* and stage *j*) of *Anopheles balabacensis* reared in the laboratory

The life expectancy for each age-stage interval (Fig. 3), used to predict the lifespan of the population, is based on the age-stage survival rate. The life expectancy (*e*_*ij*_), which is the time that an individual of age *i* and stage *j* is expected to live, appears to be almost the same for female and male adults, as indicated by their longevity (Table 1).

### Life table analysis

**Fig. 4:**
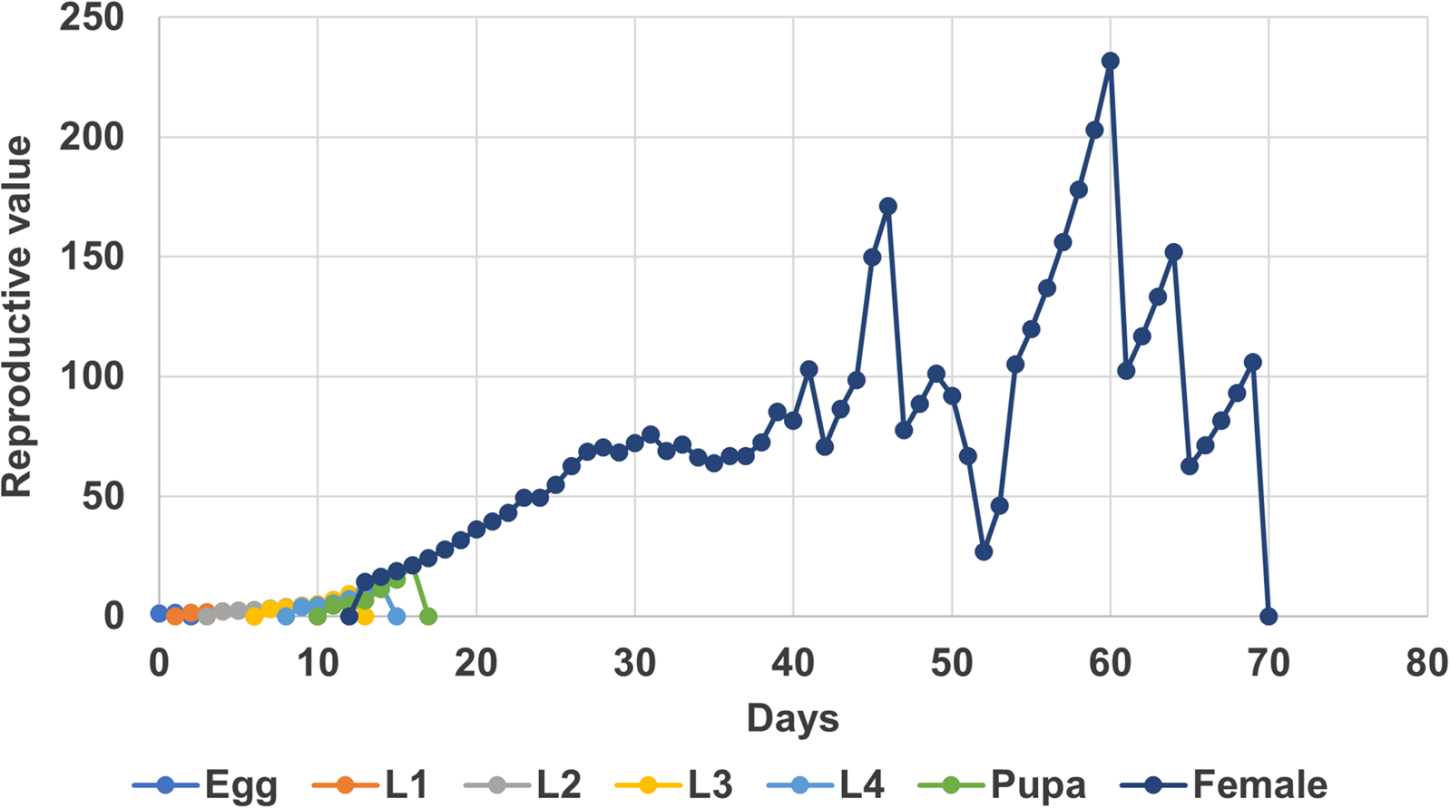
Reproductive value (*v*_*xj*_; the contribution of an individual of age *x* and stage *j* to future population growth) of *Anopheles balabacensis* reared in the laboratory

The intrinsic rate of increase (*r*) and the finite rate of increase (*λ*) are, respectively, 0.12 day^−1^ and 1.12 day^−1^, while the net reproductive rate (*R*_0_), the mean generation time and the doubling time are 46.2, 33 days and 6 days, respectively (Table 3). The reproductive value of female adults starts on day 15 (Table 3) and ends on day 69 (Fig. 4).

**Table 3:** Population parameters (mean ± SE) of *Anopheles balabacensis* reared in the laboratory

Net reproductive rate^a^ (*R*_0_)	46.21 ± 14.97
Intrinsic rate of increase^b^ (*r*)	0.12 ± 0.01
Finite rate of increase^c^ (*λ*)	1.12 ± 0.01
Mean generation time (*T*; days)	33.02 ± 1.85
Fecundity^d^	163.91 ± 39.09
Doubling time	5.97
The lowest age (days) of reproductive value of female adults	15

The means and SEs were estimated using the jackknife method with 100,000 repetitions embedded in the software

^a^Average number of offspring a female produces in her lifetime

^b^Number of progeny born to each female mosquito per unit of time

^c^Rate of increase per individual per unit time

^d^Physiological maximum potential reproductive output of a female over her lifetime

## Discussion

We present here, to the best of our knowledge, the first life table data on *An. balabacensis*, the main vector of *P. knowlesi* in Sabah. The values of* r*, *λ*,* R*_0_ and* T* were, respectively, 0.12 day^−1^, 1.12 day^−1^, 46.2 and 33 days.

Under the laboratory conditions used here, together with an adequate supply of food and water of good quality, the survival rates of the immatures were, as expected, high. For example, the hatching rate and the survival rate of the larvae at stages I-IV were 0.96 or more. However, it is unlikely that such high rates occur in the field, as female mosquitoes usually lay eggs in temporary water pools in tyre imprints or animal footprints, which may have limited nutrients and dry out before the adults emerge. The total duration of adult emergence from the egg stage was about 14.5 days in the laboratory, which may be shorter than that under field conditions. For example, the first-instar larvae of *Anopheles albimanus* in habitats in forested areas took more than 20 days to develop into adults [30]. Data on *An*. *balabacensis* can be integrated with meteorological and land cover change data to assess impacts of deforestation on the population dynamics of this vector.

A range of values of both* R*_0_ and* r* have been reported in the literature for *Anopheles* spp. [26-28, 30, 36] (Table 4). *Anopheles albimanus* and *Anopheles vestitipennis* have the highest reported* R*_0_ (ca. 300), while *An. balabacensis* has the lowest (46), which is less than half of that of *An. gambiae* (105). For *An. arabiensis*, *R*_0_ was higher in the dry season (70) than in the rainy season (54), and lower under simulated spring conditions (51) compared to summer conditions (80). The value of* r* is lowest for *An. balabacensis* (0.12 day^−1^) and highest for *An. albimanus* (0.32 day^−1^), followed by that for *A. gambiae* (0.29 day^−1^). The generation time is shortest for *An. gambiae* (5.4 days), and when coupled with its high* R*_0_ (105), might indicate potentially high population growth in this species. All these species show characteristics of* r*-strategists, in that the adults have relatively high* r* and* R*_0_ but a short generation time, while the immature stages grow rapidly and have high mortality.

**Table 4:** Comparison of life table parameters of *Anopheles* species

Species	Net reproductive rate (*R*_0_)	Instantaneous rate of increase (*r*; day^−1^)	Generation time (*T*)	Rearing conditions	References
*Anopheles balabacensis*	46.20 ± 14.97	0.12 ± 0.01	33.0	Laboratory	Present study
*Anopheles arabiensis*	70.13 ± 5.44	0.17 ± 0.01	25.0	House in a lowland deforested area; dry season	[30]
	54.29 ± 3.79	0.16 ± 0.01	25.0	House in a lowland deforested area; rainy season	
*An. arabiensis*	50.60	0.09	43.6	Laboratory; reared at 23.2 °C (spring temperature)	[27]
	79.55	0.17	25.7	Laboratory; reared at 26.4 °C (summer temperature)	
*Anopheles gambiae*	104.90 ± 28.00	0.29 ± 0.01	5.4	Laboratory; susceptible strain, 13th generation	[36]
*Anopheles albimanus*	309.20	0.32	18.0	Laboratory	[26]
	297.00	NA	NA	Laboratory	[28]
*Anopheles vestitipennis*	302.00	NA	NA	Laboratory	[28]

Both* R*_0_ and* r* are good indicators of population growth, and in insects are affected by environmental variables such as temperature, shade, amount of available food, type of food, and intraspecific competition due to crowding, etc. For example, a study conducted in houses within deforested sites showed that the larval-to-adult survivorship of *An. arabiensis* increased to 65–82% [29], and the larval-to-adult development time was shortened by 8–9 days [30]. Similarly the* R*_0_ and* r* values of *An. arabiensis *in deforested areas were 2.7–3.6 and 1.4–1.5 times more than those in forested areas [30]. However, in the present study, where the food supply was adequate, water quality good and the temperature constant, the values of* r*, *λ*,* R*_0_ and* T*, which were respectively 0.12 day^−1^, 1.12 day^−1^, 46.2 and 33 days, seem to indicate good population growth. We do not have data from the field, but nevertheless would expect lower values there. Future studies could also assess the impacts of microclimatic conditions of different habitat types.

The age-stage, two-sex life table approach was chosen for this analysis of *An. balabacensis* as it considers the age-stage structure of a population, and thus keeps track of the overlap between stages, as depicted in the graph in which s_*xj*_ is plotted (Fig. 1). Variations in the duration of immature development are also reflected in the survival and fecundity curves. Many previously published works on the life table of insects were conducted using female data only [30, 37]. The two-sex approach takes into consideration the important contribution of males to population growth, as the sex ratio and mating opportunities affect this population parameter. This approach also eliminates the possibility of erroneous* l*_*x*_ curves, as observed in analyses where only female age-specific data were considered [35]. An age-stage, two-sex life table can provide more complete and accurate information on changes in stage structure during population growth. Understanding stage structure is important in modelling because survival rates of immatures vary with stage.

The data generated from the age-stage, two-sex life table analysis could be used to quantitatively simulate the effects of variable reproduction rates (e.g. due to various environmental conditions) on the population size and stage structure of *An. balabacensis*, and to evaluate an action threshold of the vector for control measures used to reduce the population size in computer modelling. Epidemiological modelling of simian malaria requires good estimates of population parameters such as* S*_*xj*_,* m*_*x*_,* f*_*xj*_,* l*_*x*_,* R*_0_, and* r*. Life tables can provide comprehensive information on the growth rate of a population, and can be an important tool in evaluating and comparing population fitness under differing conditions when using modelling.

A Ross–MacDonald-type model has been published that compares the plausibility of transmission scenarios with variable rates of human–vector–human transmission [38]. The model appears to mainly explore how the spatial distribution of hosts and vectors affects the transmission of the disease, and includes very little information on the biology of the vector (e.g. life cycle,* R*_0_,* r*, etc.). As noted by the authors [38], this may have been due to scarce reliable data on vector abundance and behaviour available for the model, which are required to estimate vectorial capacity. This information is particularly critical when assessing the probability of non-zoonotic transmission of *P. knowlesi*; better estimates of mosquito survival rates enable assessment of the time scale and likelihood of the parasite developing in a mosquito and being transmitted to produce a secondary case.

## Conclusions

Life table parameters of *An. balabacensis* are reported here, to the best of our knowledge, for the first time. These estimates of mosquito survival rates enable assessment of the duration and likelihood of the parasite’s development in a mosquito and its transmission to give rise to a secondary case. They can provide comprehensive information on the growth rate of a population and can be important tools in evaluating and comparing population fitness under differing conditions when using modelling. Incorporating the population parameters of *An. balabacensis* into spatial and temporal models on the transmission of *P. knowlesi* would provide better insight and increase the accuracy of epidemiological forecasting.
