# Correction: Life table analysis of *Anopheles balabacensis*, the primary vector of *Plasmodium knowlesi* in Sabah, Malaysia

**DOI:** 10.1186/s13071-022-05631-x

**Published:** 2023-01-10

**Authors:** Tock H. Chua, Benny Obrain Manin, Kimberly Fornace

**Affiliations:** 1 Department of Pathology and Microbiology, Faculty of Medicine and Health Sciences, Universiti Malaysia Sabah, Sabah, Malaysia; 2 School of Biodiversity, One Health and Veterinary Medicine, University of Glasgow, Glasgow, UK; 3 Saw Swee Hock School of Public Health, National University of Singapore, Singapore, Singapore

**Correction** **:** **Parasites & Vectors (2022) 15:442** **https://doi.org/10.1186/s13071-022-05552-9**

Following publication of the original article [1], the authors flagged that the following Acknowledgements declaration had been erroneously omitted from the article: "We wish to thank Universiti Malaysia for the research facility which enabled us to conduct the laboratory work in the Insectary located at the Faculty of Medicine and Health Sciences. The research was financed a UMS grant (GSP 001) awarded to THC and a Sir Henry Dale Fellowship awarded to KMF jointly funded by the Wellcome Trust and the Royal Society (Grant Number 221963/Z/20/Z)."

The published article has now been updated with this declaration. The authors thank you for reading and apologize for any inconvenience caused.
